# The stress responsive and morphologically regulated *hsp90 *gene from *Paracoccidioides brasiliensis *is essential to cell viability

**DOI:** 10.1186/1471-2180-8-158

**Published:** 2008-09-22

**Authors:** André M Nicola, Rosângela V Andrade, Alessandra S Dantas, Patrícia A Andrade, Fabrício BM Arraes, Larissa Fernandes, Ildinete Silva-Pereira, Maria Sueli S Felipe

**Affiliations:** 1 Department of Cell Biology, University of Brasília, Brazil

## Abstract

**Background:**

*Paracoccidioides brasiliensis *is a dimorphic fungus that causes the most prevalent systemic mycosis in Latin America. The response to heat shock is involved in pathogenesis, as this pathogen switches from mycelium to yeast forms in a temperature dependent fashion that is essential to establish infection. HSP90 is a molecular chaperone that helps in the folding and stabilization of selected polypeptides. HSP90 family members have been shown to present important roles in fungi, especially in the pathogenic species, as an immunodominant antigen and also as a potential antifungal therapeutic target.

**Results:**

In this work, we decided to further study the *Pbhsp90 *gene, its expression and role in cell viability because it plays important roles in fungal physiology and pathogenesis. Thus, we have sequenced a *Pbhsp90 *cDNA and shown that this gene is present on the genome as a single copy. We have also confirmed its preferential expression in the yeast phase and its overexpression during dimorphic transition and oxidative stress. Treatment of the yeast with the specific HSP90 inhibitors geldanamycin and radicicol inhibited growth at 2 and 10 μM, respectively.

**Conclusion:**

The data confirm that the *Pbhsp90 *gene encodes a morphologically regulated and stress-responsive protein whose function is essential to cell viability of this pathogen. This work also enforces the potential of HSP90 as a target for antifungal therapies, since the use of HSP90 inhibitors is lethal to the *P. brasiliensis *yeast cells in a dose-responsive manner.

## Background

Most cells react to temperature elevations in a stereotypical manner termed heat shock response, which is conserved from bacteria to mammals [1]. Upon heating, the cell almost completely represses transcription and translation except for a set of so-called heat shock proteins (HSPs), thus initiating its adaptation to the new environment. The induction of molecular chaperones is also seen after non-thermal stresses such as those caused by oxidative stress, low pH and treatment with cytotoxic drugs.

HSP90 is a molecular chaperone with classical *in vitro *activity of protein folding. However, unlike other molecular chaperones, *in vivo *HSP90 is not necessary for *de novo *protein synthesis; it assists only a small set of proteins, which are usually dependent on ATP-dependent HSP90 binding to perform their functions correctly [2]. HSP90 client proteins include several tyrosine and serine/threonine kinases, steroid receptors and transcription factors [3]. The HSP90 protein consists of a highly conserved N-terminal nucleotide-binding domain, a flexible charged linker and a C-terminal domain that contains a -MEEVD conserved motif responsible for binding to tetratricopeptide-repeat (TPR) proteins [4].

HSP90 family members have been shown to play important roles in fungi. In the model yeast *Saccharomyces cerevisiae*, inhibition of HSP90 by an anti-HSP90 ribozyme promotes cell lysis, indicating some potential as an antifungal therapeutic target [5]. The *Candida albicans *HSP90 has been shown to be an immunodominant antigen, both in a mouse model of infection and in human patients [6]. Based on this finding, a novel therapeutic strategy has been devised using a human recombinant antibody to HSP90, which shows intrinsic antifungal activity and synergy with amphotericin B both *in vitro *and *in vivo*. This antibody is in clinical trials with encouraging results against systemic candidiasis [7]. HSP90 immunogenicity has also been applied to a phage-displayed vaccine tested in mice, which acquired resistance to systemic *C. albicans *infection [8].

HSP90 proteins are targeted by several different pharmacologic agents; two of them, radicicol and geldanamycin, inhibit the protein's ATPase activity with great specificity and potency. Derivatives of these drugs and several novel HSP90 inhibitors are now in clinical trials for cancer chemotherapy [9]. However, cancer chemotherapy is not the only use for these drugs, as they have become useful in studies of HSP90 function. Inactivation of the *Leishmania donovani *HSP90 with geldanamycin or radicicol mimics the transmission from the vector to the mammalian host, inducing the differentiation from the insect promastigote stage towards the pathogenic mammalian amastigote stage [10]. HSP90 also influences stage differentiation in *Toxoplasma gondii*, which led to its proposition as a potential drug target [11].

*Paracoccidioides brasiliensis*, a dimorphic fungus, can be found either as a filamentous soil saprobe or as yeast cells infecting mammalian hosts. Both forms can be cultivated *in vitro *in different incubating temperatures, around 22°C for mycelium and 36°C for yeast. Mycelium cells can be transformed into yeast by raising the incubation temperature to 36°C; and then reverted to mycelium by lowering the temperature [12]. The fungus undergoes a similar morphological switch *in vivo*: the infection starts by inhalation of conidia or mycelium fragments from the environment, which then transform into yeast in the host's lungs. Pathogenicity has been intimately associated with this process, as strains unable to differentiate into the yeast form are avirulent [13]. Adaptation to different temperatures thus seems to be paramount to both fungal physiology and pathogenicity, a fact that has long elicited interest on heat shock response in such pathogens. Several *P. brasiliensis *molecular chaperones have been studied, such as MDJ1 [14], HSP60 [15], HSP70 genes [16-19] and HSP100 genes [20,21]. A boost in the knowledge of these genes came from high throughput transcriptome sequencing and expression analyses [22-24], which revealed cDNA sequences and some expression data from 48 molecular chaperones [25].

This work describes the complete cDNA characterization of the HSP90 gene from *P. brasiliensis *(*Pbhsp90*) and its expression profile, including differential expression in yeast phase, induction during mycelium to yeast transition and oxidative stress. Moreover, analysis of its role on cell viability was investigated with pharmacological inhibitors.

## Results and Discussion

**Figure 1:**
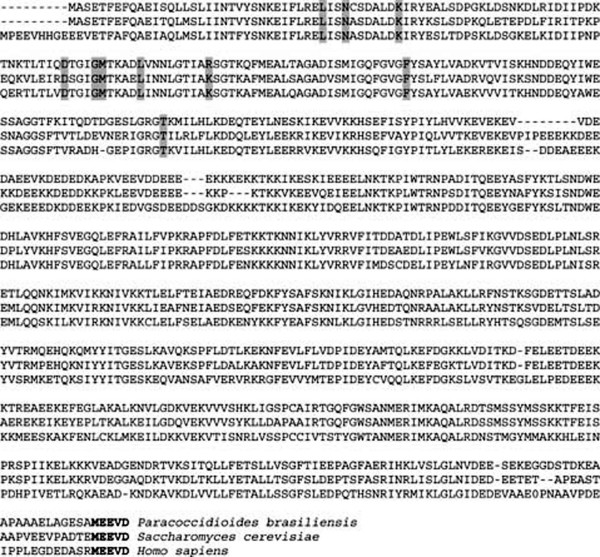
**ClustalW alignment of PbHSP90 and other HSP90 family proteins**. The *Pbhsp90 *ORF was translated and aligned with *Saccharomyces cerevisiae *(Hsp82p, accession number [GenBank: NP_015084.1]) and *Homo sapiens *(HSP90β, accession number [GenBank: AAQ63401.1]) homologues using ClustalW. Shaded amino acid residues are involved in the binding of either ATP/ADP or geldanamycin in *S. cerevisiae *[27]. The N-terminal MEEVD motif (bold) is the binding site for the TPR domain of co-chaperones. A total of 400 out of the 706 amino acid residues are conserved across the three species.

The *Pbhsp90 *cDNA was first isolated during the *P. brasiliensis *transcriptome sequencing [22,24]. In order to confirm the *hsp90 *transcript sequence, we chose a single clone which clustered in the *Pbhsp90 *transcriptome contig and fully sequenced it. The sequence shows a single open reading frame (ORF) encoding a predicted polypeptide with 706 amino acid residues that aligned with several known HSP90 proteins from organisms as diverse as fungi, plants and mammals. A search for conserved domains using NCBI rpsblast identified the Bergerat-fold N-terminal ATPase domain [26] that is typical of all HSP90 proteins. Nine out of ten residues involved in ATP/ADP and geldanamycin binding at the N-terminal domain (Leu-34, Asn-37, Lys-44, Asp-79, Gly-83, Met-84, Asn-92, Phe-124 and Thr-171) were identical in *P. brasiliensis *and *S. cerevisiae*, the only difference being a change from a lysine to an arginine at position 98 [27]. The predicted PbHSP90 protein also presents the C-terminal conserved -MEEVD motif. A total of 400 out of the 706 amino acid residues are conserved across *Homo sapiens, S. cerevisiae and P. brasiliensis *(figure 1).

**Figure 2:**
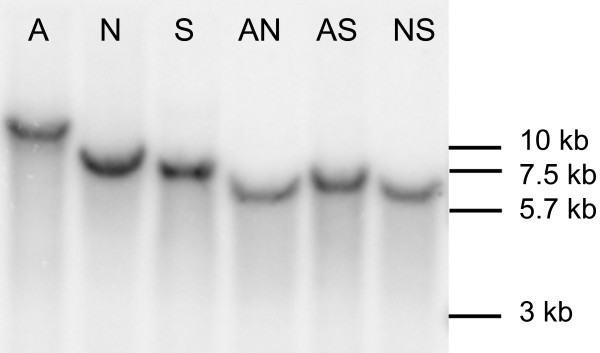
**Southern blot analysis of gene copy number**. Samples of 15 μg total DNA were digested with combinations of the restriction endonucleases ApaI (A), NdeI (N) and SacII (S), separated by agarose gel electrophoresis, blotted onto charged nylon membrane and hybridized with radioactively labelled *PbHSP90 *cDNA. Bars show molecular weight marker.

All prokaryotes and some eukaryotes are known to have a single cytoplasmic copy of the *hsp90 *gene, but some fungi present two copies [28,29]. In the later organisms, the two genes encode proteins which are structurally very similar and functionally identical. The only significant difference between the two genes is their transcriptional pattern; one is constitutively expressed and mildly induced during stress, while the other is markedly stress-induced [28]. The expression of an HSP90 gene from an organism that harbours a single copy, e.g. *C. albicans*, is somewhat like a combination of both patterns, with constitutive expression and marked stress induction of the same gene [29]. The next step in characterizing the *Pbhsp90 *gene was to evaluate the number of copies present in the genome. Southern blotting analysis using the *Pbhsp90 *cDNA as a probe has shown only one copy on the genome (Figure 2). In addition, searching the recently released genome sequence drafts from isolates Pb01, Pb18 and Pb03 [30] with the *Pbhsp90 *cDNA reveals a single copy of the gene. The translated sequence in these genomes contains three conserved introns and the predicted proteins are 98–99% identical to the one we obtained in this work.

**Figure 3:**
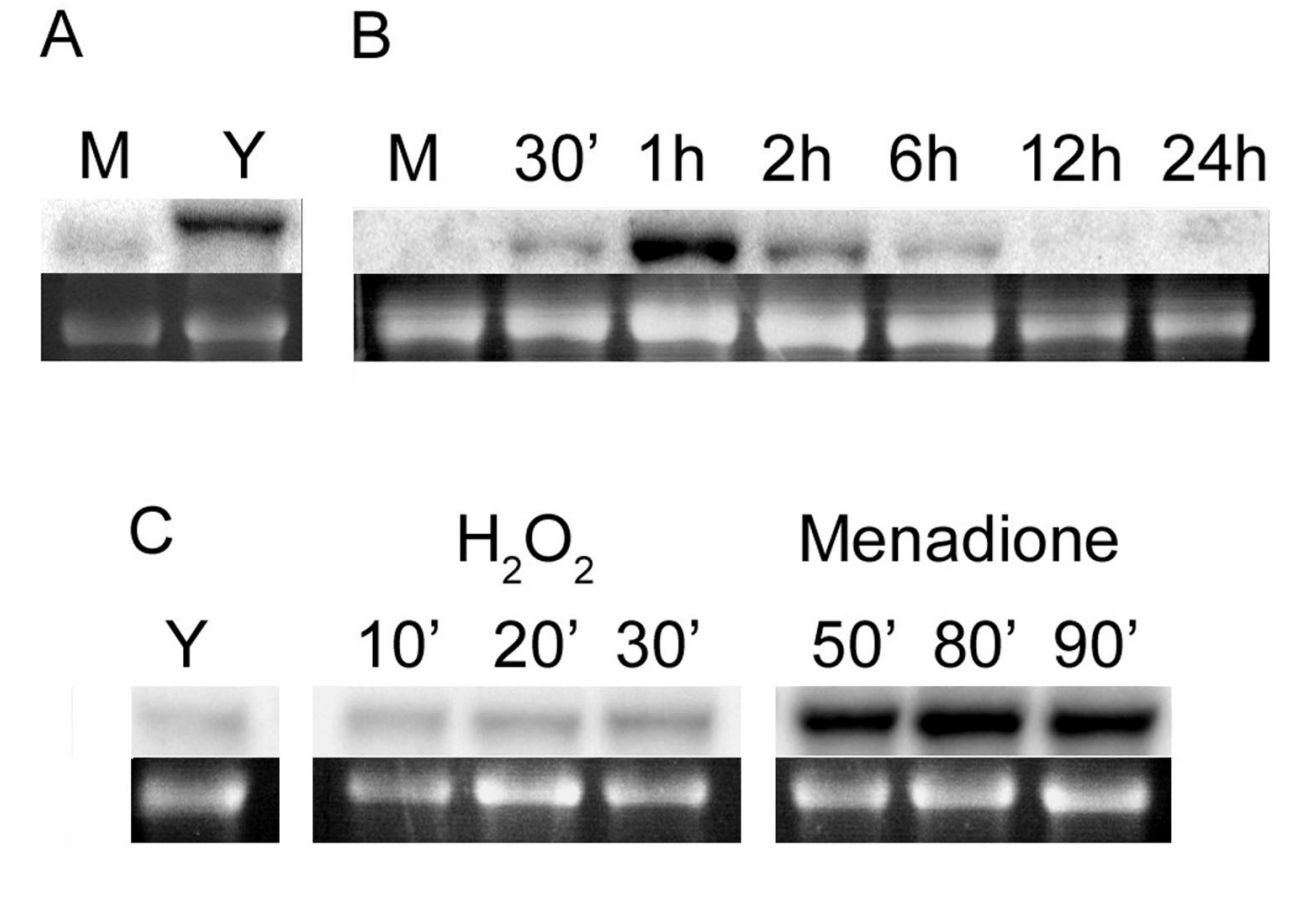
***Pbhsp90 *expression profiles in mycelium and yeast cells, during differentiation and in the presence of oxidative agents**. Total RNA (15 μg) extracted from *P. brasiliensis *was separated by denaturing agarose gel electrophoresis, blotted onto charged nylon membranes and probed with radioactively labelled *Pbhsp90 *cDNA. Panel A – mycelium (M) and yeast (Y) cells. Panel B – Mycelium cells grown at 22°C and after incubation at 36°C for up to 24 h. The membrane shown on panel B was washed to higher stringency to resolve the strong hybridization signals better. Panel C – Yeast cells incubated with 15 mM hydrogen peroxide or 1 mM menadione, a superoxide-generating reagent. In each panel, the top image represents the specific hybridization signal and the ethidium bromide stained 16S rRNA band in the bottom image.

The *Pbhsp90 *gene expression was then assessed by northern blotting under several different experimental conditions including non-stressed mycelium and yeast cultures, cells undergoing the first 24 hours of the mycelium-to-yeast transition, and oxidative-stressed yeast cells (Figure 3). As previously shown by electronic subtraction, cDNA microarray and real-time RT-PCR [22-24], expression of the *Pbhsp90 *gene can be observed in both morphological phases, but it is around 4.5 times stronger in the yeast phase (Figure 3 – panel A). These results are in accordance to what has been observed in another dimorphic fungus, *Penicillium marneffei*, in which the HSP90 protein is 5.29 times more expressed in the yeast phase [31]. The mycelium mRNA is slightly shorter than the yeast one, probably due to differences in the 3'- and/or 5'-untranslated regions (UTRs) as ESTs from mycelium and yeast formed a single cluster which corresponds to only one transcript; this has been described before for other fungal genes [32]. *Pbhsp90 *expression during the mycelium-to-yeast transition (Figure 3 – panel B) is already increased 30 minutes after the temperature shift, reaches its peak – about 25-fold relative induction – at one hour and slowly decreases during the following hours, reflecting a morphological transition thermo-dependent response [33], which is also observed by EST analysis [34]. A very similar pattern of induction has been shown for the chaperone-encoding genes *hsp60 *[15], *hsp70 *[16,18] and *hsp104 *[23].

HSP90 is known to be essential for the function of several signal transduction kinases in organisms as diverse as fruit flies [35], baker's yeast [36] and mammalians [37], including members of the MAPK and PKA families involved in fungal morphogenesis [38]. These proteins, as most in the cell after a temperature elevation of more than 10°C, are prone to denature under circumstances when signalling must occur to coordinate the transition. The very large increase on the *Pbhsp90 *mRNA levels may thus be seen not only as result of heat shock, but also as adaptation in the transition.

Another set of northern blots examined *Pbhsp90 *gene expression under some of the conditions previously shown to induce other fungal homologues. Hydrogen peroxide treatment promoted only a slight increase in *Pbhsp90 *expression, of 2.45-fold after 30 minutes (Figure 3, panel C). The H_2_O_2 _concentration used (15 mM) is far below the maximum tolerated by *P. brasiliensis*, which is close to 75 mM [39], but is three times higher than the LD_90 _for *S. cerevisiae *cells at 30 minutes [40]. Differently, two other chaperones have been shown to be induced by 5 mM H_2_O_2 _in *P. brasiliensis *[14]. In contrast to the poor induction provoked by peroxide on *Pbhsp90*, treatment with superoxide-generating menadione at 1 mM (Figure 3 – panel C) strongly induced *Pbhsp90 *expression in yeast cells. H_2_O_2 _and superoxide are among the major reactive oxygen species (ROS) used by mammalian macrophages in the defence against fungal infection [41]. *C. albicans *cells lacking Cu/Zn superoxide dismutase (SOD), a superoxide detoxification enzyme, showed increased susceptibility to macrophage attack and had attenuated virulence in mice [42]. In a review of the transcriptome sequencing project, Campos *et al*. [43] have found three catalases and three SODs in *P. brasiliensis *-isolate Pb01. The fact that superoxide radicals strongly induced *Pbhsp90 *correlates nicely with recent data from our group showing that SOD specific activity in the *P. brasiliensis *yeast cell is as strong as in non-pathogenic *S. cerevisiae *(Dantas *et al*., personal communication), while specific catalase activity is over 2000 times higher [39]. Therefore, it seems that the H_2_O_2 _detoxification by *P. brasiliensis *is so efficient that little cellular damage requiring HSP90 occurs.

**Figure 4:**
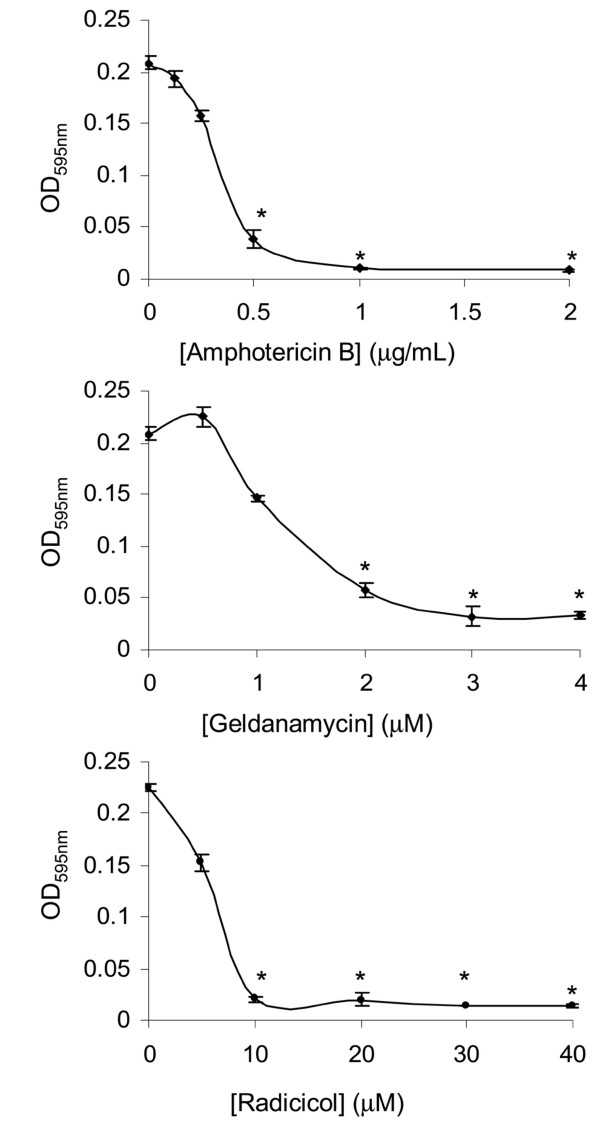
**Susceptibility of *P. brasiliensis *yeast cells to HSP90 inhibitors geldanamycin and radicicol**. Inhibition of cell growth by HSP90 targeting drugs or the control amphotericin B was tested by a broth microdilution test adapted from the international standard M27-A2. Curves show mean plus standard error of the absorbance at OD_595 nm _measured from quadruplicate experiments. *statistically significant when compared to control (p < 0.05).

It is not possible to study *Pbhsp90 *function by classical genetic approaches because knockouts of members of this family are usually not viable [28]. Furthermore, genetic manipulation is still not optimized on the multinucleated and multi-budding *P. brasiliensis *making RNA interference unfeasible as well. Those challenges have been circumvented by means of a "pharmacological knockout" with the potent and specific HSP90 inhibitors radicicol and geldanamycin. A broth microdilution test shows that both radicicol and geldanamycin inhibit the growth of *P. brasiliensis *– Pb01 yeast cells in a manner similar to amphotericin B (Figure 4), meaning that both drugs are able to penetrate *P. brasiliensis *cells and bind their targets. Moreover, it confirms that HSP90 inhibition is lethal to the cell in a dose-responsive manner and consequently that its function is essential to fungal physiology. Results similar to these have been found in other microorganisms such as *S. cerevisiae *and *Schizosaccharomyces pombe *[5,44]. The use of sub-lethal concentrations of these drugs can be foreseen as an excellent form of further investigating the broad heat shock response in *P. brasiliensis *and in special *Pbhsp90*.

## Conclusion

The data show that *Pbhsp90 *is a highly expressed gene, under complex regulation by morphological and oxidative stress signals. This picture resembles previous observations of other molecular chaperones, stressing even more the importance of heat adaptation in *P. brasiliensis *dimorphism and survival in the harsh environment inside the mammalian host. This work also enforces the potential of HSP90 as a target for novel antifungal therapies, since the use of HSP90 inhibitors is lethal to the *P. brasiliensis *yeast cells in a dose-responsive manner. Geldanamycin, radicicol and the other members of the ever-growing list of HSP90 inhibitors could be engineered to achieve higher selectiveness for the fungal protein. Taken together, these results confirm that the *Pbhsp90 *gene encodes a morphologically and stress-responsive protein whose function is essential to cell viability of this pathogen.

## Methods

### Strains and cultures

*P. brasiliensis *strain Pb01 (ATCC-MYA-826) was used throughout this study. Cells were maintained on semi-solid Fava-Netto medium, incubated at 37°C for yeast cells and 22°C for mycelia.

### cDNA sequencing and assembly

A single cDNA cloned in a Lambda ZAP phage vector (Stratagene) was excised *in vivo *according to the supplier's manual. The resulting plasmid was sequenced with vector-encoded T3 and T7 and internal primers in a MegaBACE^® ^(GE Healthcare) automatic sequencer. Base-calling, filtering and assembly were done as described before [24]. The sequence was deposited in GenBank (accession number [GenBank: AY928608]).

### Southern blot

Total DNA was obtained by phenol:chloroform extraction of mechanically disrupted frozen yeast cells. Samples of 15 μg of DNA were digested, with combinations of three restriction endonucleases (ApaI, NdeI and SacII) known not to have internal sites in the *Pbhsp90 *cDNA, and separated by agarose gel electrophoresis. The gel was blotted onto a charged nylon membrane by upward capillary transfer. The cDNA probe was amplified by PCR using vector primers. A total of 25 ng of the purified product was radioactively labelled with α-P^32^-dATP (GE Healthcare) using the MegaPrime labelling kit (GE Healthcare). The hybridized membrane was then washed as described for the northern blots below sequentially until low background was detected and then exposed to a phosphorimager, which was scanned on a Typhoon^® ^9210 scanner (GE Healthcare).

### Northern blot

RNAs from mycelium-to-yeast transition and of isolated mycelium and yeast cells were obtained as previously described [45,46]. For the oxidative stress experiments, yeast cells were washed and ressuspended in medium containing either 15 mM H_2_O_2 _or 1 mM menadione. Samples were then collected by centrifugation, frozen with liquid nitrogen and disrupted. Total RNA was then obtained by extraction with Trizol^® ^reagent (Invitrogen). Fifteen microgram samples of total RNA were separated by electrophoresis on formaldehyde-containing 1% agarose gels; blotted onto charged nylon Hybond^® ^N+ membranes (GE Healthcare) and hybridized as described for the Southern-blot. Hybridized membranes were then washed sequentially with solutions containing SDS 0.1% and different concentrations of SSPE in 20 minute rounds: 2× SSPE 50°C, 1× SSPE 50°C, 1× SSPE 65°C and 0,1× SSPE 65°C. Each membrane was monitored with a Geiger counter after each wash and exposed in a phosphorimager when the background radioactivity was low enough. Resulting bands were quantified using ImageQuant^® ^software and normalized by dividing the hybridization signal intensity by the ethidium bromide stained 18S ribosomal RNA band intensity.

### Drug susceptibility testing

Drug susceptibility tests were adapted from the international standard M27-A2 [47]. Yeast cells were grown on RPMI defined medium and diluted to a 2× suspension containing 10^5 ^cells/mL. Amphotericin B and geldanamycin were diluted in dimethyl-sulfoxide (DMSO) to 100× stock solutions; radicicol was diluted in ethanol. Working solutions (2×) were then made with RPMI medium. A 100 μL aliquot of the 2× cell suspension was added to 100 μL of the 2× drug solutions in U-shaped 96-well plates, so that the final suspension reached a cell density of 10^5 ^cells/mL and 1% DMSO or ethanol. The plate was incubated at 37°C for 7–10 days, when the minimal inhibitory concentration (MIC) was directly observed. In order to convey more precisely the observed difference in growth, OD_595 nm _was measured with a GeneQuant^® ^spectrophotometer. Each concentration was tested in quadruplicate experiments. Statistical analyses were done with one-way analysis of variance (ANOVA) and the Tamhane T2 multiple comparison test from SPSS 11.0 software.

## Authors' contributions

AMN planned and designed the study, performed the experiments and analyzed their results and drafted the manuscript. RVA, ASD and PAA prepared samples for and executed Northern blots. ASD and FBMA participated in cDNA cloning and sequencing. LF executed drug susceptibility assays and reviewed the manuscript. ISP supervised cDNA cloning, Southern and northern blots and participated on data analysis. MSSF conceived and coordinated the study, data analysis and manuscript preparation. All authors read and approved the final manuscript.
